# AFLPs and Mitochondrial Haplotypes Reveal Local Adaptation to Extreme Thermal Environments in a Freshwater Gastropod

**DOI:** 10.1371/journal.pone.0101821

**Published:** 2014-07-09

**Authors:** María Quintela, Magnus P. Johansson, Bjarni K. Kristjánsson, Rodolfo Barreiro, Anssi Laurila

**Affiliations:** 1 Dept of Animal Biology, Plant Biology and Ecology, Faculty of Science, University of A Coruña, A Coruña, Spain; 2 Animal Ecology, Department of Ecology and Genetics, Evolutionary Biology Centre, Uppsala University, Uppsala, Sweden; 3 Hólar University College, Department of Aquaculture and Fish Biology, Sauðarkrokur, Iceland; University of British Columbia Okanagan, Canada

## Abstract

The way environmental variation shapes neutral and adaptive genetic variation in natural populations is a key issue in evolutionary biology. Genome scans allow the identification of the genetic basis of local adaptation without previous knowledge of genetic variation or traits under selection. Candidate loci for divergent adaptation are expected to show higher F_ST_ than neutral loci influenced solely by random genetic drift, migration and mutation. The comparison of spatial patterns of neutral markers and loci under selection may help disentangle the effects of gene flow, genetic drift and selection among populations living in contrasting environments. Using the gastropod *Radix balthica* as a system, we analyzed 376 AFLP markers and 25 mtDNA COI haplotypes for candidate loci and associations with local adaptation among contrasting thermal environments in Lake Mývatn, a volcanic lake in northern Iceland. We found that 2% of the analysed AFLP markers were under directional selection and 12% of the mitochondrial haplotypes correlated with differing thermal habitats. The genetic networks were concordant for AFLP markers and mitochondrial haplotypes, depicting distinct topologies at neutral and candidate loci. Neutral topologies were characterized by intense gene flow revealed by dense nets with edges connecting contrasting thermal habitats, whereas the connections at candidate loci were mostly restricted to populations within each thermal habitat and the number of edges decreased with temperature. Our results suggest microgeographic adaptation within Lake Mývatn and highlight the utility of genome scans in detecting adaptive divergence.

## Introduction

Temperature influences all physiological processes from the molecular level to that of the whole organism [1], [2], affects individual fitness and exerts a profound impact on the structure, dynamics and functioning of populations [2]–[4]. Because of these influences, the way organisms adapt to thermal variation has long captivated the attention of scientists. In the last decades, the study of thermal adaptation has evolved from studies focusing on simple tolerance towards those dealing with mechanisms shaping the temperature-dependent performance and the functional optimum of species [5]. Studies focusing on physiological variation among wild populations exposed to different thermal conditions improve our knowledge of how populations adapt to different thermal environments. Traditionally, thermal adaptation has been studied by investigating clinal variation in physiological and fitness traits along thermal gradients, in combination with reciprocal transplants (e.g. [6], [7]) or common garden experiments (e.g. [7]–[10]); but the recent advances in molecular tools are now allowing us to disentangle the evolutionary processes behind thermal adaptation.

Population genomics has become an increasingly useful approach to detect adaptive genetic divergence in nonmodel species see [11]. Genome scans allow the identification of loci with atypical levels of genetic differentiation (e.g. [12], [13]). These outlier loci are potentially under directional selection (e.g. [14]–[17]) and the distribution of neutral and non-neutral genomic regions among contrasting thermal environments can aid understanding of the evolutionary role of thermal selection and neutral evolutionary processes. Identifying the way in which neutral genomic regions and regions putatively under thermal selection are distributed among thermal environments provides insights into the genetic properties that allow and constrain thermal adaptation.

The distribution of genetic variation within and among populations of a species is determined by the interaction between natural selection and neutral evolutionary processes such as drift and gene flow. While evolutionary change can be very fast with measurable genetic change occurring over only a few generations [18], [19], the pace of evolution and its final outcome depends on several factors such as genetic diversity [20], [21], ecological and evolutionary costs associated with adaptive change, and interactions with other evolutionary forces. Specifically, gene flow and genetic drift may disturb the effects of divergent selection and prevent local adaptation (e.g. [22]–[26]). However, local adaptation can evolve in the presence of gene flow when local selection is strong (e.g. [27]–[30]), and can itself constrain gene flow through selection against maladapted immigrants [31].

There is a long tradition of research on local adaptation in freshwater and terrestrial habitats [32], [33], and more recently in the marine environment see [7]. In pond snails, a number of studies have found local adaptation, especially in terms of antipredator responses affecting shell morphology and behaviour (e.g. [34]–[36]). Similarly, thermal adaptation has been found in directly developing marine gastropods at both microgeographic (0.05–15 Km) [37]–[39] and macrogeographic (400->3500 Km) [40]–[43] scales.

Lake Mývatn, a shallow eutrophic freshwater lake situated in an area of active volcanism in northern Iceland, provides an intriguing system to study thermal adaptation. The lake is subjected to geothermal activity with several warm (up to 30°C) and cold (ca. 5°C) springs running in the lake. In the areas between these springs, water temperature follows the seasonal fluctuations. Hence, the Lake Mývatn system consists of patches of low or high temperatures, which are separated by large areas where temperature fluctuates seasonally [44]. As fluctuations in water temperature in the vicinity of the springs are small, aquatic life close to the outflow of cold and warm water can be expected to have adapted to these stable temperature environments.

In this paper, our objective was to investigate if the freshwater gastropod *Radix balthica* showed signs of fine-grained thermal adaptation in this system with strong temperature contrasts. For this purpose we used AFLP markers and several outlier detection procedures to quantify the proportion of the scanned genome likely to be under divergent selection, and compared the patterns of neutral and adaptive divergence between the thermal environments. In addition, we tested the association between temperature and frequencies of AFLP markers and COI haplotypes by logistic regression as implemented in the Spatial Analysis Method (SAM) [45], and conducted network analyses [46] to assess how geographic distance and temperature structure neutral and adaptive genetic variation. AFLPs were the marker of choice because, despite of being dominant, they are highly reproducible and show advantages such as low development and genotyping efforts in contrast with other more modern markers such as SNPs [47].

## Materials and Methods

### Study system

Lake Mývatn was created by a large basaltic lava eruption some 2300 years ago [48]. For a detailed description of the ecology of Lake Mývatn, see Einarsson *et al*. [44]. The common pond snail *Radix balthica* is a wide-spread directly-developing aquatic pulmonate snail belonging to the Clade Hygrophila (Family Chilinidae Dall, 1870) [49] and distributed throughout North-Western Europe. It occupies slow-flowing rivers, littoral zones of lakes and ponds [50] and has a relatively wide tolerance of pH, salinity and temperature, but prefers calcareous waters [51]. As a hermaphrodite it is capable of both self- and cross-fertilization, but is considered to be a preferential outcrosser [52]. It has usually one generation per year.

### Sampling, AFLP genotyping, and COI sequencing

**Figure 1:**
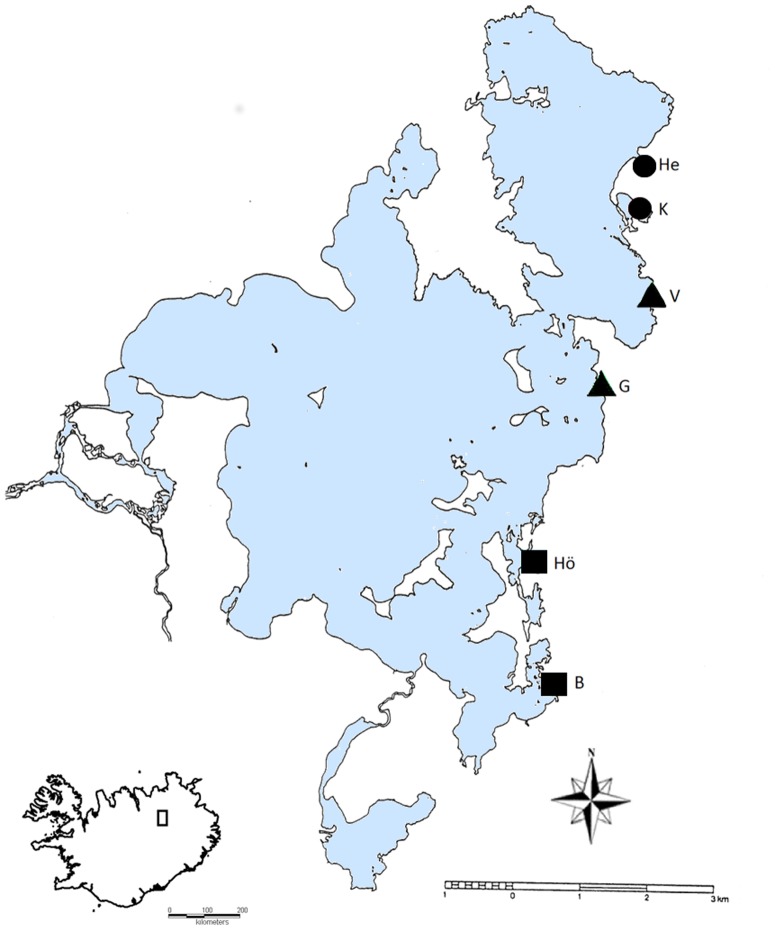
Map of Lake Mývatn (Iceland) showing the localization of the area of study (water body colored). Circles depict sites at high temperature (24°C); triangles show sites at intermediate temperatures (12–19°C), and squares correspond to sites at low temperature (6–8°C). See the text for further information.

We sampled *R. balthica*, at six sites in Lake Mývatn on 20th and 21st June 2009. Being this species is common and widespread in Iceland; no official permits are required to conduct sampling. However, the snails used in this study were collected with the permission from the local landowners. During the time of collection, surface water temperature at these sites, measured with a portable thermometer, ranged from 6 to 24°C (Fig. 1) and represented the entire range of thermal environments within the lake. Water temperatures were also measured at the same locations between 18 May and 18 September 2011 with three iButton thermochrons (DS1921G-F50) placed at the depth of ca. 50 cm each site. While the temperatures measured at cold (B and Hö) and warm (He and K) locations in 2009 reflect the stable temperatures near the cold (2011: 7.01°C±0.58; 6.39°C±0.70) and warm springs (2011: 22.96°C±1.40; 22.20°C±1.08), respectively; populations G (2009: 12°C; 2011: 8.89°C±2.38) and V (2009: 19°C; 2011: 8.59°C±2.59) are subjected to wide seasonal temperature fluctuations with water temperature ranging between 1 and 20°C (note that the mean temperatures for the seasonal sites above are lower than average due to exceptionally cold weather in late May and June 2011).

In the cold and warm locations the snails were collected in the immediate vicinity of the springs. Samples of 30 individuals per site were collected and stored in 70% ethanol at −20°C until processing. Animals were dissected under a stereomicroscope and a piece of the mantle was removed for DNA extraction. To avoid cross contamination, each specimen was dissected using disposable scalpels and tweezers that were flame-sterilized between individual samples. Genomic DNA was isolated using Qiagen DNeasy Blood & Tissue isolation kit (Qiagen Inc., Valencia, CA) following the manufacturer's instructions.

AFLPs were performed following the *Tru1I*/EcoRI protocol of Vos *et al*. [53]. Fifteen selective primer combinations producing 376 repeatable loci (13–38 per primer pair) were selected for the study. The reproducibility of AFLP profiles was assessed by duplicate independent DNA from 10% of the individuals (three per population). Furthermore, six extractions with no tissue were included to obtain a “template” for the blanks. See Supporting Information for further details.

A 643-bp region of the mitochondrial COI gene was amplified in all the individuals using the universal primers described by Folmer *et al*. [54]. Amplifications were performed in 25 µl of a solution containing 0.5 µM of each primer, 0.2 mM of each dNTP, 2 mM MgCl_2_, 1x AmpliTaq buffer, 1.25 U AmpliTaq DNA polymerase (Applied Biosystems) and 1.5 µl DNA. Cycling conditions were 2 min denaturing at 95°C followed by 30 cycles of 30 s at 95°C, 30 s at 45°C, and 1 min at 72°C. After removing the excess of primers and nucleotides (with shrimp alkaline phosphatase and exonuclease I enzymes), samples were sequenced by Macrogen Inc. (Macrogen, Seoul, South Korea) on ABI 3730 instruments (Applied Biosystems). Sequences were checked and edited using the program ChromasPro (Technelysium Pty. Ltd., Eden Praire, MN) and aligned using BioEdit [55] with default settings. After alignment and trimming, the final sequence length was 643 bp. jModelTest 0.1 [56] identified HKY+I model as the best-fit model of DNA evolution. Calculations of genetic diversity indices were performed in DnaSP version 5.10 [57]. The sequences of the 25 distinct COI haplotypes were deposited in GenBank under accession numbers JN624782–JN624806.

### AFLPs: Detection of loci under selection

Two different basic approaches allow screening for loci that are putatively under selection: (i) methods for detecting outlier loci that show unexpectedly high or low differentiation [58]–[60]; and (ii) correlative methods that look for associations between genetic variation and environmental variables [61]–[63].

To minimize the risk of detecting false positives, we used three different procedures to detect loci with a pattern deviating from neutrality. First, we used the hierarchical Bayesian method described in Beaumont & Balding [64] as implemented in BayeScan software [59]. This method is based on the principle that genetic differentiation among populations living in contrasting environments is expected to be different for loci under selection than for the rest of the genome. BayeScan estimates population-specific F_ST_ coefficients and uses a cut-off based on the mode of the posterior distribution [59]. The program was run by setting sample size to 10000 and the thinning interval to 50 as suggested by Foll & Gaggiotti [65], resulting in a total chain length of 550000 iterations. The loci with a posterior probability over 0.99 were retained as outliers, corresponding to a Bayes Factor >2 (*i.e.* “decisive selection” [61]) and providing substantial support for the model.

Secondly, we used the Fdist approach by Beaumont & Nichols [58] implemented in Mcheza [66]. Loci with an unusually high F_ST_ are putatively under directional selection, while loci with low F_ST_ value are considered to be potentially under stabilizing selection. We simulated the neutral distribution of F_ST_ with 100000 iterations at a significance level of 0.01. This method also implements a multitest correction based on false discovery rates (FDR) that is fundamental to avoid high overestimation of the percentage of outliers (*e.g*. 1% of false positive with a threshold of 99%).

Finally, we used the Spatial Analysis Method (SAM) described by Joost *et al*. [62] based on evaluation of the incidence of spatial or environmental coincidence. SAM identifies alleles associated with environmental variables without the need of populations to be defined. However, it must be combined with the abovementioned methods to differentiate between directional and stabilizing selection. SAM calculates logistic regressions between all possible marker-environmental pairs and determines if a model including an environmental variable is more informative than a model including only the constant. We tested for the effect of temperature under a restrictive approach, which considered a model significant only if both G and Wald Beta 1 tests rejected the corresponding null hypothesis at the 0.0000505 confidence level after Bonferroni correction, corresponding to *P* = 0.01.

BayeScan, Mcheza and SAM were used conservatively with the analyses restricted to the loci with a dominant allele frequency between 5% and 95% across the whole dataset. The rationale behind this was to decrease the probability that differentiation, at a given locus, would be mistakenly identified as a signature of selection merely because it stood out against low levels of background genetic variation resulting from the inclusion of low-polymorphism markers.

### AFLPs: Genetic structure

After outlier detection, the original data set was partitioned into two subsets: loci under selection and neutral loci. The subset of neutral loci was produced by retaining the loci that were regarded as non-selected by all three outlier detection methods. Allele frequencies in the neutral data set were estimated using the Bayesian method of Zhivotovsky [67] implemented in *AFLP-SURV* v1.0 [68] under the option of non-uniform prior distributions of allele frequencies. Overall F_ST_ was calculated with *AFLP-SURV* both for neutral loci and for loci under selection, and significance was based on 10000 permutations. Genetic differentiation among populations was estimated separately for neutral and non-neutral data sets by the Analysis of Molecular Variance, AMOVA [69] implemented in the program GeneAlEx v6.4 [70].

To investigate population structure regardless of environmental variation or geographic distance, we identified genetic clusters in the AFLP-neutral dataset with the Bayesian model-based clustering algorithms implemented in STRUCTURE v. 2.3.3 [71]–[73] under a model assuming admixture and correlated allele frequencies without using population information. Ten runs with a burn-in period of 100000 replications and a run length of 1000000 Markov chain Monte Carlo (MCMC) iterations were performed for a number of clusters ranging from K 1 to 10. We then applied the Evanno *et al*. [74] *ad hoc* summary statistic ΔK which is based on the rate of change of the ‘estimated likelihood’ between successive K values. Furthermore, we compared the posterior probabilities for the values of K with the highest P(X|K) using a Wilcoxon two-samples test according to Rosenberg *et al*. [75]. Runs of the selected K were averaged with CLUMPP version 1.1.1 [76] using the LargeKGreedy algorithm and the G′ pairwise matrix similarity statistics and results were visualized as a barplot. However, STRUCTURE algorithm may be poorly suited for inferring the number of genetic clusters in data sets characterized by isolation-by-distance relationships [71], [74] and, also given that it puts a strong prior on the existence of clusters, it may be prone to errors when geographical sampling is discrete along clines [77]. Therefore we also performed this analysis using TESS, which has been reported to be superior to other Bayesian clustering methods under certain conditions [77]. Thus, the MCMC algorithm was run for the AFLP-neutral dataset under CAR admixture model with interaction parameter W = 0.6, 10000 burn-in and 50000 sweeps. Some 50 independent iterations were run for K = 1–6 and after identifying the value of K that produced the highest likelihood, this was ran 100 times and the 20 highest likelihood runs were averaged using CLUMPP version 1.1.1 [76] applying the LargeKGreedy algorithm and the G′ pairwise matrix similarity statistics. Results were visualized as a barplot.

### AFLPs: Population Graphs

We performed network analyses to investigate network structure and genetic connectivity among sites. Graph theory is an area of mathematics that deals with problems of connectivity and flow of networks, with proven utility in population genetics and landscape ecology see [46], [78]. A graph represents a landscape of discrete habitat patches as a set of nodes (sites) genetically interconnected by edges (gene flow) between them [79]. The presence of an edge in a Population Graph is determined by the genetic covariance of the connected populations. If these are independent, conditional on the remaining data in the model, the populations are not connected. We applied a multivariate population graphing approach [46], [78] to compare the genetic networks generated by neutral and non-neutral markers.

Networks were constructed using the online application Population Graphs v2 (http://dyerlab.github.io/popgraph/. Accessed 2014 June 16), and the analyses were performed with the software Genetic Studio [80]. For graph construction, we retained the minimal edge set that sufficiently described the total among-population covariance structure. Two sites will share an edge if there is significant genetic covariance between them after removing the covariance that each population has with all the remaining populations in the network. Significance was evaluated using edge exclusion deviance, which identifies the most important edges for each node in terms of genetic covariance. For a full mathematical and statistical description of genetic methods for constructing graphs see Dyer & Nason [46] and references therein. A binomial test for the existence of two subgraphs within the dataset [46], [81] was used for each network to determine whether there was restricted gene flow between the populations from high and low temperature. In partially reproductively isolated populations, we would expect to find two subgraphs for the loci under directional selection but not for the neutral ones. Across the entire Population Graph, graph distance was estimated as the minimal topological distance connecting pairs of sites (nodes). The two pairwise graph distances matrices obtained (for neutral markers and markers under selection, respectively) were then regressed on the shortest geographic distance within the lake to test for Isolation-By-Graph Distance (IBGD). This test allows us to detect the existence of genetic barriers to gene flow among populations. Assuming a homogeneous IBD process, graph distances and spatial distances should be proportional, but the relationship between expected edge length and spatial distance may change if migration is heterogeneous. A relatively small graph distance between spatially distant sites indicates long-distance migration (extended edges). Conversely, geographical or ecological barriers that impede migration relative to other localities with similar distance generate relatively high graph distances (compressed edges: [82]). Correlations and detection of extended and compressed edges were determined by regressing geographic and Graph Distance [80].

Neutral and adaptive genetic variation distributed across space are net products of different evolutionary mechanisms (genetic drift, gene flow and selection), and it is important not to confound these components and the mechanisms shaping their spatial distribution. To evaluate whether genetic drift may explain the spatial genetic patterns found in neutral markers, we performed a Mantel [83] test between the matrices of genetic distance (estimated as pairwise F_ST_) and geographical distance (*i.e.* shortest distance by water), expecting to find Isolation-by-Distance (IBD) due to restricted gene flow. Similarly, a correlation with thermal distance would produce an Isolation-by-Temperature (IBTe) pattern. To detect a signature of selection and thereby local adaptation, we correlated the genetic distance of non-neutral loci with geographic distance and performed this analysis also using Partial Mantel tests with covariance of neutral pairwise genetic distance as a third matrix. Mantel and Partial Mantel tests were conducted with PASSaGE [84] and significance was tested after 10000 permutations. A similar approach was used to substitute F_ST_ with Graph Distance to test for the existence of Isolation-By-Graph Distance (IBGD) and Isolation-By-Graph Distance-Temperature (IBGDTe) patterns (results in Table H in File S1).

### mtDNA: Population structure and correlation between haplotype frequencies and temperature

Hierarchical AMOVA was used to test population structure with COI haplotype frequencies using Arlequin version 3.5.1.2 [85]. Pairwise Φ_ST_s were calculated and their significance was based on 10000 permutations. In an identical approach to the one conducted with AFLPs, SAM was used to investigate the correlation between haplotype frequencies and environmental variables. The haplotypes that showed positive correlation with the temperature were considered to be of particular importance for adaptation to temperature. Likewise, population graphs were conducted separately on mtDNA haplotypes that were related to temperature and on those that were not. The correlations of genetic distances over geographical and thermal distances for all pairs of populations were tested with the Mantel and partial Mantel permutation procedures with PASSaGE as explained above for AFLPs. These tests were also performed regressing Graph Distance against geographic and thermal distance (in Supporting Information).

The evolutionary relationships between haplotypes were examined with the software Network 4.6.1.0 (http://fluxus-engineering.com/accessed 2014 Jun 14) using the median-joining algorithm (epsilon set to 10) and the connection cost distance method to build an unrooted cladogram [86]. Transversions were weighted three-fold to transitions because they are rarer in mitochondrial DNA. Two hypermutable sites were identified by post-processing using the Steiner (MP) algorithm, and their weight was halved (i.e. set to 5). The MJ network was maximum parsimony post-processed to display the shortest tree.

## Results

### AFLPs: Selection tests

198 of the 376 repeatable AFLP loci had dominant allele frequencies ranging between ≥5% and ≤95%, and were included in the outlier analyses. BayeScan identified ten loci exceeding the threshold for “very strong” evidence of selection (log_10_BF>1.5), none of them under stabilizing selection. Six out of the ten loci under directional selection met our more stringent criterion for evidence of “decisive” selection (log_10_BF>2) and were incorporated to the non-neutral data set. In contrast, the neutral data set was composed of the loci that did not reach the threshold of “substantial” evidence of selection, *i.e.* loci with log_10_BF<0.5. Diversity statistics for all markers and neutral markers alone can be found in Table A in File S1.

Four out of the six markers that showed log_10_(BF)>2 in BayeScan were identified as being under directional selection with Mcheza at 99% confidence (Table B in File S1). This approach also identified two additional loci under directional selection as well as eight loci under stabilizing selection.

After calculating logistic regressions between all possible marker-environment pairs (a total of 198 models), and significance threshold set to 99% (corresponding to P<0.00005), SAM detected 60 loci associated with temperature. This set of 60 markers also included the ten outlier loci classified by BayeScan as above “very strong selection” (*i.e.* log_10_(BF)>1.5) as well as the six loci identified by Mcheza (Table C in File S1). The analyses of the four extreme populations shown in Table C in File S1 reported signs of selection in four of the markers identified in the analyses of the six total populations.

The final neutral data set consisted of 117 markers that were identified as neutral by all three outlier detection methods. Likewise, the final data set of loci under directional selection consisted of the four loci that were detected by all three approaches. The population wise frequency distributions for the four markers under directional selection (Table D in File S1) showed that C18_143 and C34_277 were linked to high temperature environments, whereas C34_282 was mainly present in low temperature populations.

### AFLPs: Genetic structure

Most of the variation detected by AMOVA for the set of 117 neutral markers was found among individuals within populations (88%); however, population differentiation was still highly significant (F_ST_ = 0.119, *P*<0.0001). For the set of four loci under directional selection, the picture changed as the distribution of the variance within populations decreased to 45% with more than four times higher levels of genetic structuring among populations (F_ST_ = 0.546, *P*<0.0001). The set of eight loci under stabilizing selection showed no variance among populations (F_ST_ = 0.000, *P* = 0.976).

Pairwise F_ST_ estimates (Table E in File S1) based on the 117 neutral AFLP markers ranged from 0.043 to 0.131, all of them significantly different from zero at 1% confidence, whereas for loci under directional selection they showed a general increasing trend with increasing thermal differences between populations (ranging between 0.007^NS^ and 0.774***).

**Figure 2:**
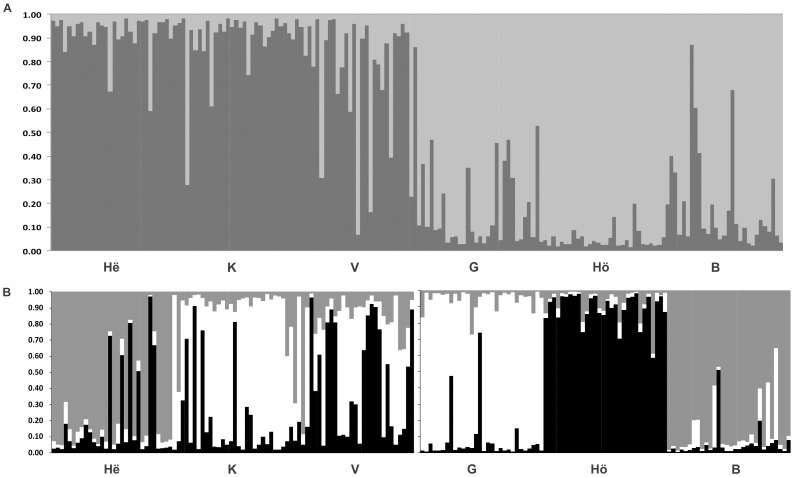
Hierarchical Bayesian clustering for the six populations genotyped at 117 neutral loci. First (upper barplot, Fig. 2A), inferred ancestry of individuals was calculated after averaging ten STRUCTURE [71]–[73] runs with CLUMPP [76]. The number of clusters that best fits the total data was K = 2 after Evanno's [74] correction (ΔK = 306). Then (lower barplots, Fig. 2B), the same procedure was performed again for every cluster obtaining K = 3 for each of the subsets (ΔK = 58 and ΔK = 242 for high and low temperature respectively). Sites are ordered in the barplots according to the N-S directions that corresponds to decreasing habitat temperatures.

Applying the method of Evanno *et al.* [74] to STRUCTURE runs, strong evidence (ΔK = 306) was found to assign the six populations to two clusters following the thermal differences (Fig. 2, upper barplot) with a clear discontinuity in the proportion of membership to each cluster in the intermediate zone. Thus, population V (19°C) joined the cluster of high temperature whereas population G (12°C) clustered with the cold-temperature group. Next, the same procedure was performed on each cluster separately according to the hierarchical approach. In both clusters, evidence was found to assign the individuals to three different clusters (ΔK = 58 and ΔK = 242 for high and low temperature respectively) (Fig. 2, lower barplots). Populations in the lower range of temperature showed more distinct features with lesser levels of admixture than the ones at higher temperature.

TESS revealed K = 6 as the most likely number of clusters, thus supporting STRUCTURE hierarchical approach results and neutral pairwise F_ST_. After averaging the 20% best TESS runs with CLUMPP, a variable degree of admixture was detected in all the populations (Fig. A in File S1). The highest similarity was found between K and V, which was coherent with these populations showing the lowest yet significant pairwise F_ST_ (0.0433***).

Mantel tests (Table 1) showed strong and positive correlation between F_ST_ and geographic distance, revealing IBD at AFLP neutral markers (r = 0.611, *P* = 0.022) and at loci under directional selection (r = 0.864, *P* = 0.003). The same pattern was found when considering the thermal habitats, and hence both neutral markers (r = 0.684, *P* = 0.009) and loci under directional selection (r = 0.917; *P* = 0.008) showed statistically significant IBTe. The relations found when regressing F_ST_ *vs*. geographic or thermal distance remained largely similar when testing F_ST_ against Graph Distance (Table H in File S1). The only exception was the neutral markers *vs*. geographic distance, which was found to be marginally non-significant (*P* = 0.0617, Table H in File S1) and thus not showing Isolation-by-Graph Distance (IBGD).

**Table 1:** Results on Mantel and partial Mantel tests comparing matrices of geographic distance (Geo), temperature (Temp) and genetic distance as pairwise F_ST_ obtained for AFLPs and COI for neutral markers (NF_ST_); for loci under directional selection (DirF_ST_) and for the loci under stabilizing selection (StaF_ST_).

	Mantel tests			Partial Mantel tests		
Molecular marker	Matrices	Mantel's r	*P*-value	Matrices	Mantel's r	*P*-value
**AFLPs**	Geo-NF_ST_	**0.6112**	**0.0217**	Geo-NF_ST_(Temp)	0.0686	0.4285
	Geo-DirF_ST_	**0.8641**	**0.0029**	Geo-DirF_ST_ (Temp)	0.3835	0.2135
				Geo-DirF_ST_(NF_ST_)	**0.7726**	**0.0351**
	Geo-StaF_ST_	NAN	1.0000	Geo-StaF_ST_(Temp)	NAN	1.0000
				Geo-StaF_ST_(NF_ST_)	NAN	1.0000
	Temp-NF_ST_	**0.6838**	**0.0088**	Temp-NF_ST_(Geo)	0.3927	0.1405
	Temp-DirF_ST_	**0.9174**	**0.0075**	Temp-DirF_ST_(Geo)	0.6833	0.0739
				Temp-DirF_ST_(NF_ST_)	**0.8389**	**0.0494**
	Temp-StaF_ST_	NAN	1.0000	Temp-StaF_ST_(Geo)	NAN	1.0000
				Temp-StaF_ST_(NF_ST_)	NAN	1.0000
COI mtDNA	Geo-NF_ST_	0.0350	0.3246	Geo-NF_ST_(Temp)	0.1239	0.2977
	Geo-DirF_ST_	**0.7638**	**0.0146**	Geo-DirF_ST_(Temp)	0.0554	0.3881
				Geo-DirF_ST_(NF_ST_)	**0.7860**	**0.0066**
	Temp-NF_ST_	−0.0340	0.4880	Temp-NF_ST_(Geo)	−0.1237	0.4581
	Temp-DirF_ST_	**0.8763**	**0.0186**	Temp-DirF_ST_(Geo)	0.6667	0.1043
				Temp-DirF_ST_(NF_ST_)	0.8871	0.0194

Boldface type indicates significant values after 9999 permutations. NAN = “not a number”.

NOTE: COI DirF_ST_ refers to the haplotypes correlated with temperature whereas COI NF_ST_ refers to the uncorrelated ones.

### AFLPs: Network properties

**Figure 3:**
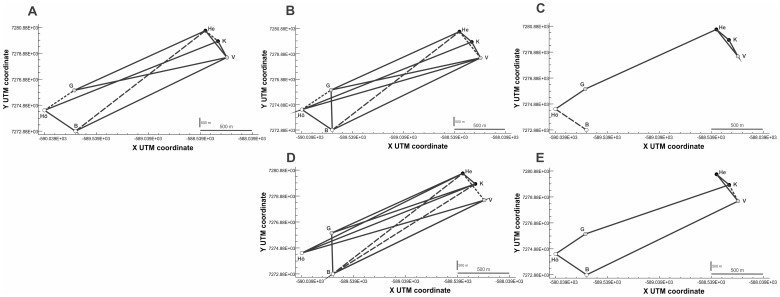
Graphs of the genetic networks for AFLPs (upper row) and COI (lower row). Nuclear networks have been created with: a) eight loci under stabilizing selection, b) 117 neutral loci and c) four loci under directional selection; whereas mitochondrial networks have been created with d) 22 uncorrelated haplotypes and e) three haplotypes associated with temperature. Black circles represent populations in high temperature sites (24°C), grey circles depict intermediate temperatures (12–19°C) and white circles represent sites at low temperature (6–8°C). Site pairs in a network connected by lines are considered to exchange migrants exhibiting significant conditional genetic covariance. Solid lines indicate connections assuming an IBD process where genetic distances and spatial distances are proportional. Dotted lines (…) represent compressed edges with relatively higher conditional genetic distance (cGD) in respect to spatial distance (suggesting geographical or ecological barriers), whereas dashed lines (---) depict extended edges indicating long distance migration processes.

The network that best depicted the behaviour of the neutral markers data set (Fig. 3b) contained edges connecting almost all the populations with each other (12 edges out of the 15 possible ones). Topology generated by loci under stabilizing selection (Fig. 3a) showed a similar morphology but with a slightly less dense net of connections. In contrast, in the network for loci under directional selection, not only was the total number of connections halved, but there was only one edge connecting contrasting thermal habitats (Fig. 3c). A Mantel test revealed the existence of IBGD for markers under directional selection (r = 0.565, *P* = 0.012), but this was not the case for neutral markers (r = 0.189, *P = *0.076) nor for markers under stabilizing selection (r = 0.123, *P* = 0.164).

The network generated by neutral markers showed connections within and between habitats (Fig. 3b). The connections between contrasting habitats accounted for 67% of proportional edges and 33% of extended ones, whereas within similar habitats, 67% of connections were proportional and 33% compressed. Likewise, the stabilizing network (Fig. 3a) showed connections within and between habitats: 60% of edges between contrasting habitats were proportional and 40% extended, whereas 60% of edges between similar habitats were proportional and 40% compressed. Loci under directional selection showed proportional connections among all the populations and only one extended edge joining the sites at the lowest temperature (Fig. 3c).

When testing the possibility that population structure was composed of distinct genetic groups according to the habitat type, the null hypothesis stated that the probability of obtaining an edge connecting both subgraphs in the graph was 0.4 (binomial test). Within graphs, a significant deficiency of edges between “high” and “low temperature” habitat sites, hence depicting two subgraphs, was found for loci under directional selection (*p*(*X*≤ *K*_between high and low temperature_) = 0.041), but not for neutral markers (*p* = 0.335) nor for loci under stabilizing selection (*p* = 0.367) (Table 2).

**Table 2:** Results of the analyses searching the presence of two subgraphs in the network determined by high and low temperature for AFLP markers and COI haplotypes.

Loci	Network	Bridge probability between habitats	Separating Edge Set between habitats	p(X≤ Kbth)	Full Graph |Nodes|	Full Graph |Edges|	Edge probability
**AFLPs**	Stabilizing selection	0.6	5	0.367	6	10	0.4
	Neutral	0.6	6	0.335	6	12	0.4
	Directional selection	0.6	1	**0.041**	6	6	0.4
**COI**	Uncorrelated to temperature	0.6	8	0.881	6	11	0.4
	Correlated to temperature	0.6	2	0.096	6	7	0.4

Analyses were performed with the module Graph of the software Genetic Studio.

### mtDNA: Population structure and correlation between haplotype frequencies and temperature

The 174 individuals successfully sequenced for COI showed 23 mutations in 22 polymorphic sites (8 of them parsimoniously informative), which generated 25 different haplotypes within a range of 10 km. Three of these haplotypes correlated with temperature according to SAM (Table F in File S1), suggesting that they may be of importance for temperature adaptation. Allele frequencies for Hap_15 showed a clear cline of increasing frequency with decreasing temperature starting from 19°C, whereas the frequencies of Hap_4 and Hap_6 showed a tendency to decrease with decreasing temperature.

An AMOVA was performed separately for the two sets of haplotypes. At uncorrelated haplotypes, 9.25% of the genetic variation was due to differences among populations with an average fixation index significantly different from zero (Φ_ST_ = 0.093, *P* = 0.002). At haplotypes correlated with temperature, variation among populations was seven times higher (64.65%, Φ_ST_ = 0.646, *P*<0.0001).

The pairwise Φ_ST_ at haplotypes correlated with temperature (Table G in File S1) ranged from 0.000^NS^ to 0.903*** and showed values significantly different from zero in all pairwise comparisons between contrasting habitats as well as in the pair He vs. V (both high temperature).

Genetic differentiation measured as pairwise neutral Φ_ST_ did not follow a pattern of IBD (r = 0.035, *P* = 0.325) nor IBTe (r = −0.034, *P* = 0.488) when regressed over geographic or thermal differences, respectively (Table 2). However, haplotypes related to temperature showed both IBD (r = 0.764, *P* = 0.015) and IBTe (r = 0.877, *P* = 0.019). Partial Mantel tests only showed positive IBD and IBTe for haplotypes associated with temperature when corrected for uncorrelated haplotype Φ_ST_. The only controversy with the Mantel tests performed with Graph Distance occurred for haplotypes not associated with temperature, where the IBGD pattern was inverted (r = −0.573, *P* = 0.011, Table H in File S1).

Population graphs revealed a very dense network of connections (11 edges) for uncorrelated haplotypes (Fig. 3d) depicting intense gene flow, even among populations from different thermal habitats (8 edges). As mentioned above, this set of markers followed an inverted pattern of IBGD with genetic differentiation decreasing with geographic distance. By contrast, the topology generated by the three haplotypes related to temperature (Fig. 3e) yielded 7 edges, only two of them connecting contrasting habitats, and IBGD significantly explained the genetic differentiation among populations (r = 0.696, *P = *0.026). The graph was not divided into subgraphs in any of the cases (Table 2), although the probabilities for subdivision were highly different between the marker types *(P* = 0.881 for unassociated haplotypes and 0.096 for haplotypes associated with temperature).

The star-like haplotype network (Fig. C in File S1) was consistent with a historical expansion of the population size. The three dominant haplotypes (Hap1, Hap4 and Hap15) were located in the centre of the star with 16 out of 22 remaining haplotypes differing from them by one single nucleotide substitution. Four haplotypes (7, 9, 12, and 14) differed by two mutational steps.

## Discussion

We found evidence for divergent selection among *R. balthica* populations with ca. 2% of the loci under directional selection, a proportion that fits within the 2–5% intervals reported for similar AFLP genome scans in different taxa [14]–[16], [87], [88]. Our results suggest fine-grained adaptation to local thermal conditions and emphasize the potential of combining information from neutral and candidate loci with ecological data to identify contrasting patterns of genetic variation related to different environmental conditions. These findings add to the growing body of recent literature that has compared spatial patterns of neutral markers and loci under selection to unravel the effects of gene flow, genetic drift and selection (e.g. [16], [17], [87], [89]–[92]).

### Microgeographic thermal adaptation in *Radix balthica*?

We found contrasting patterns for neutral markers and loci under directional selection with an average differentiation at the former ones (either AFLPs or COI) at least four times lower than at those under directional selection. However, there were remarkable discrepancies between the marker types suggesting that geographic distance may affect them differently. The patterns of population connectivity revealed by network analyses were similar for neutral AFLP markers and COI haplotypes showing intense gene flow among populations, even between contrasting thermal environments (Fig. 2b, d). Alternatively genetic drift may be stronger for mitochondrial markers because the effective population size is only 1/4 that of the nuclear markers. The lack of division into subgraphs indicated that the distribution of neutral genetic variation was unrelated to thermal variation.

When comparing populations from contrasting thermal habitats, the average F_ST_ found for loci under directional selection was 0.549 at AFLPs and 0.722 at COI, indicating that selection can maintain higher levels of population differentiation at these loci than in the neutral ones. Furthermore, the network analyses showed that the patterns of population connectivity were qualitative and quantitatively different from the neutral topologies, with connections mostly restricted to populations within the same thermal habitat (Fig. 2c, e). The number of edges connecting divergent thermal habitats was restricted to one (AFLPs) or two (COI), suggesting that temperature is a strong environmental axis that conditions the genetic composition of the populations at both thermal extremes. Thus, the distribution of the edges was in agreement with former studies reporting relationships between habitat features and functional genetic variation e.g [15], [17], [93]–[95]. Likewise, two subgraphs were detected for AFLPs (*P* = 0.041), although there was only nonsignificant indication for two subgraphs in COI (*P* = 0.096).

The fact that neutral and candidate markers depicted different graph patterns suggests either no or limited gene flow at genomic regions under directional selection, and supports the idea of local thermal adaptation in the face of ongoing gene flow. This was indicated by 1–2 edges connecting high and low temperature habitats in the topology of loci under directional selection, which sharply contrasted with the 6–8 edges found either in the neutral topology or in the topology of loci under stabilizing selection. Directional selection on fitness traits can be strong enough to maintain divergence in these genomic regions by reducing the effective recombination in genes affecting local adaptation, but does not reduce recombination in other genomic regions [17]. For instance, Kavanagh *et al.* [21] and Junge *et al.* [96] provided evidence that natural selection is sufficiently powerful for freshwater fish populations to adapt to novel temperature regimes within 22 generations, even under conditions of low genetic variation and under the influence of gene flow. The logical step for future research would be, firstly, the characterization of anonymous outlier AFLP markers to detect any eventual underlying genes with the further aim to associate candidate genes under putative selection and experimental phenotypic data. Experiments measuring thermal performance and tolerance of genotyped individuals could confirm whether these genetic polymorphisms play a role in thermal adaptation.

The evidence for divergent selection between different thermal habitats in *R. balthica* at a scale of less than 10 km agrees with former studies that have found thermal adaptation in ranges of 0.05–15 km in directly developing marine gastropods [37]–[39].

While our analyses clearly support thermal adaptation in *R. balthica*, a pair of issues needs further consideration. Firstly, the spatial distribution of loci under directional selection was influenced also by spatial distance as significant IBGD was found both for AFLPs (r = 0.565, *P* = 0.012) and COI (r = 0.458, *P = *0.025). The explanation for this is most likely the strong correlation between thermal differentiation and geographic distance (r = 0.8559, *P* = 0.0027), which hampers the efforts to tease apart the contribution of these two factors. There may also be other, co-varying factors affecting this correlation such as water chemistry [97], but further studies are needed to elucidate this. Secondly, the accurate detection of outliers is not free from difficulties, one of them being that different procedures yield different results. In our case, both BayeScan and Mcheza (the latter merging DFDIST kernel with a user-friendly interface) reported the same number of loci under directional selection, although only four of them were detected by both methods. This situation is similar to the one described by Nunes *et al.* [87] with DFDIST and BayeScan detecting a similar proportion of outliers (3–4%), but only a few of them being detected by both methods. Caballero *et al.* [98] raised several concerns about the sensitivity of DFDIST, and Pérez-Figueroa *et al.* [99] performed a simulation study to compare the efficiency of DFDIST, DETSELD and BayeScan to detect loci under directional selection with dominant markers, concluding that BayeScan was more effective under a wide range of scenarios. However, when the ultimate aim of the genome scan is to target candidate loci influenced by selection for further research, it is advisable to combine several methods to increase the power of the search despite increasing Type I error rate.

In addition, a noteworthy feature in the discrepancies between outlier detection approaches was that although both BayeScan and Mcheza reported six markers to be under divergent selection (four of them common) and almost identical strength of correlations for IBD and IBTe (not shown), the network approach provided a completely different picture of the relationships and connections between populations (Table H, Fig. Ba,c in File S1). This highlights the utility of network analyses as a tool that might allow disentangle how evolutionary processes have acted on interacting populations [46], while complementing the picture provided by traditionally used methods such as pairwise F-statistics, IBD models and spatial autocorrelations.

### Non-neutrality of mtDNA

The use of SAM outlier detection procedure allowed us to identify three mitochondrial haplotypes seemingly linked to different thermal habitats (Table F in File S1), which yielded a strongly significant pattern of IBTe. Hap_15 showed the clearest trend of increasing allele frequency with decreasing temperature starting at 19°C. By contrast, Hap_6 was present in high frequency at 24°C but declined to zero at 12°C, and Hap_4 was not present in the two coldest locations. To our knowledge, outlier detection methods have not been formerly used on mitochondrial DNA; however, mitochondrial haplotype frequencies strongly associated with environmental variables have been reported in a variety of systems (e.g. [31], [100], [101]).

As SAM only detects an association between habitat type and haplotype, our results can be considered preliminary and leave a lot of room for alternative, nonadaptive explanations. However, it is interesting to note that while evolutionary and population genetic studies have traditionally assumed that sequence variation in mtDNA undergoes neutral or nearly neutral evolution [102], the important roles of all thirteen mtDNA encoded peptides in cellular ATP production suggest that mtDNA variation can have significant metabolic and fitness consequences [103]. Indeed, a growing body of evidence indicates that the assumption of neutrality may not be valid, e.g [104] and references therein. In this context, several authors have advocated the idea that mtDNA and mito-nuclear gene complexes might evolve adaptively by selection imposed from the prevailing thermal environment [105]–[107]. The rationale behind is that mtDNA encodes multiple subunits in four of the five respiratory enzyme complexes [103], [108] and that enzymatic processes are temperature sensitive. Thus, adaptation to a novel thermal environment might result in selection for gene products with different thermal properties [107]. Furthermore, COI is a protein encoding gene and selection on it could be expected. However, the lack of recombination in mtDNA complicates this scenario: with no recombination, the mitochondrial genome is particularly susceptible to genetic hitchhiking accompanying selection at linked sites, and allele frequencies may rarely be at the stationary neutral distribution [102]. In addition, although statistical analyses of DNA sequences suggest that evolution of mitochondrial proteins is not always neutral, the functional significance of variation in these proteins has not been extensively explored and biochemical and physiological studies are required.

Finally, available data suggest that the driving force behind evolutionary change is not always adaptation to the external environment. Subtle genetic changes introduced into a population's gene pool by mutation face constant selection favoring the maintenance of functional interactions among proteins encoded by nuclear and mitochondrial genes. Hence, natural selection on mtDNA-encoded peptides simultaneously results both in adaptation to the environment and co-adaptation to the nuclear genome [103].

In conclusion, the comparison of spatial patterns of neutral markers and loci under selection among populations of the gastropod *Radix balthica* living in contrasting environments highlights the usefulness of complementing genome scans for selection in natural populations with ecological data to disentangle the effects of gene flow, genetic drift and selection. Our results suggest microgeographic adaptation within Lake Mývatn and highlight the utility of genome scans in detecting adaptive divergence.

## Data Archiving

**DNA sequences**: Genbank accessions JN624782–JN624806

**AFLPs data**: DRYAD entry doi:10.5061/dryad.3nd75

## Supporting Information

File S1 **Supporting Information.** Supporting information contains detailed description of the AFLPs reaction together with the three figures (Fig. A-C) and eight tables (Table A-H). **Figures:** **Fig. A**. Histograms of assignment probabilities calculated by TESS for the six populations genotyped at 117 neutral loci after averaging the 20% best runs for K = 6 with CLUMPP [5]. Each vertical bar represents an individual and its assignment proportion into six clusters. Sites are ordered in the barplot according to the N-S directions that corresponds to decreasing habitat temperature. **Fig. B.** Graphs of the AFLPs genetic networks created with a) six loci under directional selection detected by BayeScan with log_10_(BF)>2 (“very strong selection”); b) 60 loci under directional selection detected by SAM, and c) six loci under directional selection detected by Mcheza. Black circles represent populations in high temperature sites (24°C), grey circles depict intermediate temperatures (12–19°C) and white circles represent sites at low temperature (6–8°C). Site pairs in a network connected by lines are considered to exchange migrants exhibiting significant conditional genetic covariance. Solid lines indicate connections assuming an IBD process where genetic distances and spatial distances are proportional. Dotted lines (…) represent compressed edges with relatively higher conditional genetic distance (cGD) in respect to spatial distance (suggesting geographical or ecological barriers), whereas dashed lines (---) depict extended edges indicating long distance migration processes. **Fig. C.** Median-joining network of COI haplotypes. Each haplotype is represented by a circle, and its area is proportional to its relative frequency; shared haplotypes are represented as frequency diagrams. Smaller black circle (mv) represents an unsampled hypothetical haplotype. Numbers correspond to mutational positions in the studied 643-bp fragment. Colors in the diagrams follow a decreasing thermal trend (i.e. warmest populations are depicted in the darkest colors). **Tables:** **Table A.** Diversity statistics by population for 376 repeatable AFLP markers, using Bayesian estimation of allele frequencies with non-uniform priors [6]. Numbers in parenthesis correspond to the values for the 117 neutral loci found simultaneously with BayeScan, Mcheza and SAM. **Table B.** Shaded cells with numbers in bold depict loci detected to be under directional selection by: SAM (P values for G and Wald Beta 1 with a significance threshold set to 95% corresponding to P<0.000103093 after Bonferroni correction); BayeScan (log_10_(BF)>1.5 corresponding to “very strong selection”) and MCHEZA at a significance P value of 0.01. **Table C.** Analysis of four populations at extreme temperatures.- Shaded cells with numbers in bold depict loci detected to be under directional selection by: SAM (P values for G and Wald Beta 1 with a significance threshold set to 95% corresponding to P<0.000103093 after Bonferroni correction); BayeScan (log_10_(BF)>1.5 corresponding to “very strong selection”) and MCHEZA at a significance P value of 0.01. The markers selected in the analyses with the total six populations are depicted in bold italics. **Table D.** Distribution of frequencies (%) per population for the four markers under directional selection detected with the three approaches (BayeScan, Mcheza and SAM) simultaneously. **Table E.** Pairwise F_ST_ between populations calculated with AFLPSurv for the 117 neutral data (below diagonal) and pairwise F_ST_ for the four loci under directional selection (above diagonal). Pairwise F_ST_ for loci under stabilizing selection took value of 0.000^NS^ for every pair of populations. Significance was based on 10000 permutations. **Table F.** mtDNA. Distribution of frequencies (%) per population for the haplotypes showing correlation with temperature according to SAM. **Table G.** Pairwise Φ_ST_ between populations calculated with the 22 haplotypes not correlated with temperature (below diagonal) and with the 3 correlated haplotypes (above) computed from haplotype frequencies frequencies with Arlequin. Significance was based on 10000 permutations. **Table H.** Results on Mantel and partial Mantel tests comparing matrices of geographic distance (Geo), temperature (Temp) and genetic distance (estimated as pairwise Graph Distance with GeneStudio) for AFLPs and COI, and assessed for: neutral markers (NGD); loci under directional selection (DirGD) and loci under stabilizing selection (StaGD). Boldface type indicates significant values after 9999 permutations.
(DOCX)
