# Supplementary material for: AFLPs and Mitochondrial Haplotypes Reveal Local Adaptation to Extreme Thermal Environments in a Freshwater Gastropod
Source: PLoS One. 2014 Jul 9;9(7):e101821. doi: 10.1371/journal.pone.0101821 (PMC4090234; doi:10.1371/journal.pone.0101821)
Supplement: File S1 — Supporting Information. Supporting information contains detailed description of the AFLPs reaction together with the three figures (Fig. A-C) and eight tables (Table A-H). Figures: Fig. A. Histograms of assignment probabilities calculated by TESS for the six populations genotyped at 117 neutral loci after averaging the 20% best runs for K = 6 with CLUMPP [5]. Each vertical bar represents an individual and its assignment proportion into six clusters. Sites are ordered in the barplot according to the N-S directions that corresponds to decreasing habitat temperature. Fig. B. Graphs of the AFLPs genetic networks created with a) six loci under directional selection detected by BayeScan with log10(BF)>2 (“very strong selection”); b) 60 loci under directional selection detected by SAM, and c) six loci under directional selection detected by Mcheza. Black circles represent populations in high temperature sites (24°C), grey circles depict intermediate temperatures (12–19°C) and white circles represent sites at low temperature (6–8°C). Site pairs in a network connected by lines are considered to exchange migrants exhibiting significant conditional genetic covariance. Solid lines indicate connections assuming an IBD process where genetic distances and spatial distances are proportional. Dotted lines (…) represent compressed edges with relatively higher conditional genetic distance (cGD) in respect to spatial distance (suggesting geographical or ecological barriers), whereas dashed lines (---) depict extended edges indicating long distance migration processes. Fig. C. Median-joining network of COI haplotypes. Each haplotype is represented by a circle, and its area is proportional to its relative frequency; shared haplotypes are represented as frequency diagrams. Smaller black circle (mv) represents an unsampled hypothetical haplotype. Numbers correspond to mutational positions in the studied 643-bp fragment. Colors in the diagrams follow a decreasing thermal trend (i.e. warmest [file pone.0101821.s001.docx]

**FILE S1. Supporting Information**

**AFLPs and mitochondrial haplotypes reveal local adaptation to extreme thermal environments in a freshwater gastropod**

María Quintela, Magnus P. Johansson, Bjarni K. Kristjánsson, Rodolfo Barreiro, Anssi Laurila

**MATERIAL AND METHODS**

**AFLP reactions**

We used several control measures to guarantee the reproducibility of our AFLP fingerprints. First, we tested 56 combinations of primers with three selective bases on 8 individuals (4 from high temperature and 4 from low temperature). The whole procedure was repeated with new, independent DNA extractions of the same individuals to check for reproducibility. All the combinations managed to consistently amplify across the sample set so care was taken to choose those ones who produced the easiest to score panels as well as the most polymorphic ones. Therefore, 15 combinations generating reproducible, easily scorable profiles were chosen to perform the study. Samples and blanks were randomized in the plates using the sample function implemented in R-package [[1](#_ENREF_1)].

AFLP reactions were performed following the protocol of Vos *et al.* [[2](#_ENREF_2)]. Briefly, DNA extractions from each individual were diluted in TE buffer to a final concentration of 24-35ng/µl. 10µl of which were restricted with 2.5 units of *EcoRI* and *Tru1I* in a total volume of 20µl containing 2X Tango buffer (Fermentas). After incubation the product was added to 6µl of ligation solution containing 0.43μM *EcoRI*-adapters (5'-CTCGTAGACTGCGTACC-3' and 5'-AATTGGTACGCAGTCTAC-3'), 4.3Μm *Tru1I*-adapters (5'-GACGATGAGTCCTGAG-3' and 5'-TACTCAGGACTCAT-3'), 0.52 units of T_4_ ligase (Fermentas) and 10X ligation buffer in order to create a particular binding site for the primers at the ends of the restriction fragments obtained. Once the product was diluted 10-fold in Milli-Q H_2_O (Millipore Co.), 10µl were used for the preselective amplification with 0.3μM E-primers with the T selective nucleotide (5'-gactgcgtaccaattct-3'), 0.3μM T-primers with the C selective nucleotide (5'-gatgagtcctgagtaacac-3'), 2.5mM MgCl_2_, PCR buffer, 0.04µg/µl BSA, 0.2μM dNTPs and 0.04 units of *AmpliTaq* polymerase (Applied Biosystems) that yielded a first subset of fragments while improved the ground for the next amplification. Ten µl from the 10-fold diluted product from this preamplification were finally used for the selective amplification with 0.6μM of primer E and 0.6μM of primer T, both of them with 3 extra selective nucleotides that match into the fragments, 0.8μM dNTPs, 2.5mM MgCl_2,_ 0.04μg/μl BSA, PCR buffer, and 0.4 units *AmpliTaq Gold* polymerase (Applied Biosystems). The preamplification was performed immediately after ligation, whereas the products of the other reactions were kept overnight at -20ºC. PCR reactions were performed in a Hybaid thermocycler model PXE. The 5' end of E-primers was labeled with FAM or NED fluorescence. During reactions, individuals, negative controls and blind samples were randomly placed in PCR plates. All dilutions were prepared in laminar flow cabin; all DNA and PCR product solutions were added using filter tips. DNA fragments were separated on a 3130xl Genetic Analyzer (Applied Biosystems) and those ranging from 40 to 400 bp were manually scored for presence/absence at each selected locus with the help of GeneMarker v.1.70 (SoftGenetics LLC, State College, PA, USA) with the suggested options for AFLP. All the electropherograms per combination were gathered to generate an automatic panel of markers; panels that were purified following the common recommendations [[3](#_ENREF_3),[4](#_ENREF_4)] and used to translate each electropherogram into a binary matrix (presence/absent of bands). Those less reproducible markers were removed using as reference the pairs of replicated individuals.

The final primer combinations (EcoRI/TruI) retained were: -TAG/-CGT; -TAG/-CAC; -TAG/-CTA; -TAG/-CTT; -TAG/-CTG; -TAG/-CAA; -TCT/-CAG; -TGC/-CAC; -TGC/-CTA; -TGC/-CTT; -TCA/-CAG; -TAC/-CTA; -TAC/-CTG; -TGG/-CAC; -TGG /-CTT.

AFLP products were coded with a two digits number consisting first on the number of the *EcoRI* primer (1: Eco-TAG, 2: Eco-TCT, 3: Eco-TGC, 6: Eco-TCA, 7: Eco-TAC, 8: Eco-TGG, 9: Eco-TCC); and secondly the number of the *Tru1* primer (1: Tru-CAG, 2: Tru-CGT, 3: Tru-CAC, 4: Tru-CTA, 5: Tru-CAT, 6: Tru-CTT, 7: Tru-CTG, 8: Tru-CAA) followed by the size of the fragment in bp (*e.g.* C34_125, meaning “code 34 (Eco-TGC_Tru-CTA)_125 bp”).

**REFERENCES**

1. Team RDC (2008) R: A language and environment for statistical computing. Vienna: R Foundation for Statistical Computing.

2. Vos P, Hogers R, Bleeker M, Reijans M, Lee vdT, et al. (1995) AFLP: a new technique for DNA fingerprinting. Nucleic Acids Research 23: 4407-4414.

3. Bonin A, Pompanon F, Taberlet P (2005) Use of amplified fragment length polymorphism (AFLP) markers in surveys of vertebrate diversity. In: Zimmer E, Roalson E, editors. Molecular Evolution: Producing the Biochemical Data, Part B. San Diego, CA: Academic Press. pp. 145-161.

4. Whitlock R, Hipperson H, Mannarelli M, Butlin RK, Burke T (2008) An objective, rapid and reproducible method for scoring AFLP peak-height data that minimizes genotyping error. Molecular Ecology Resources 8: 725-735.

5. Jakobsson M, Rosenberg NA (2007) CLUMPP: a cluster matching and permutation program for dealing with label switching and multimodality in analysis of population structure. Bioinformatics 23: 1801-1806.

6. Zhivotovsky LA (1999) Estimating population structure in diploids with multilocus dominant DNA markers. Molecular Ecology 8: 907-913.

**FIGURES**

|  |
| --- |

Fig. A. Histograms of assignment probabilities calculated by TESS for the six populations genotyped at 117 neutral loci after averaging the 20% best runs for K=6 with CLUMPP [[5](#_ENREF_5)]. Each vertical bar represents an individual and its assignment proportion into six clusters. Sites are ordered in the barplot according to the N-S directions that corresponds to decreasing habitat temperature.

| a) 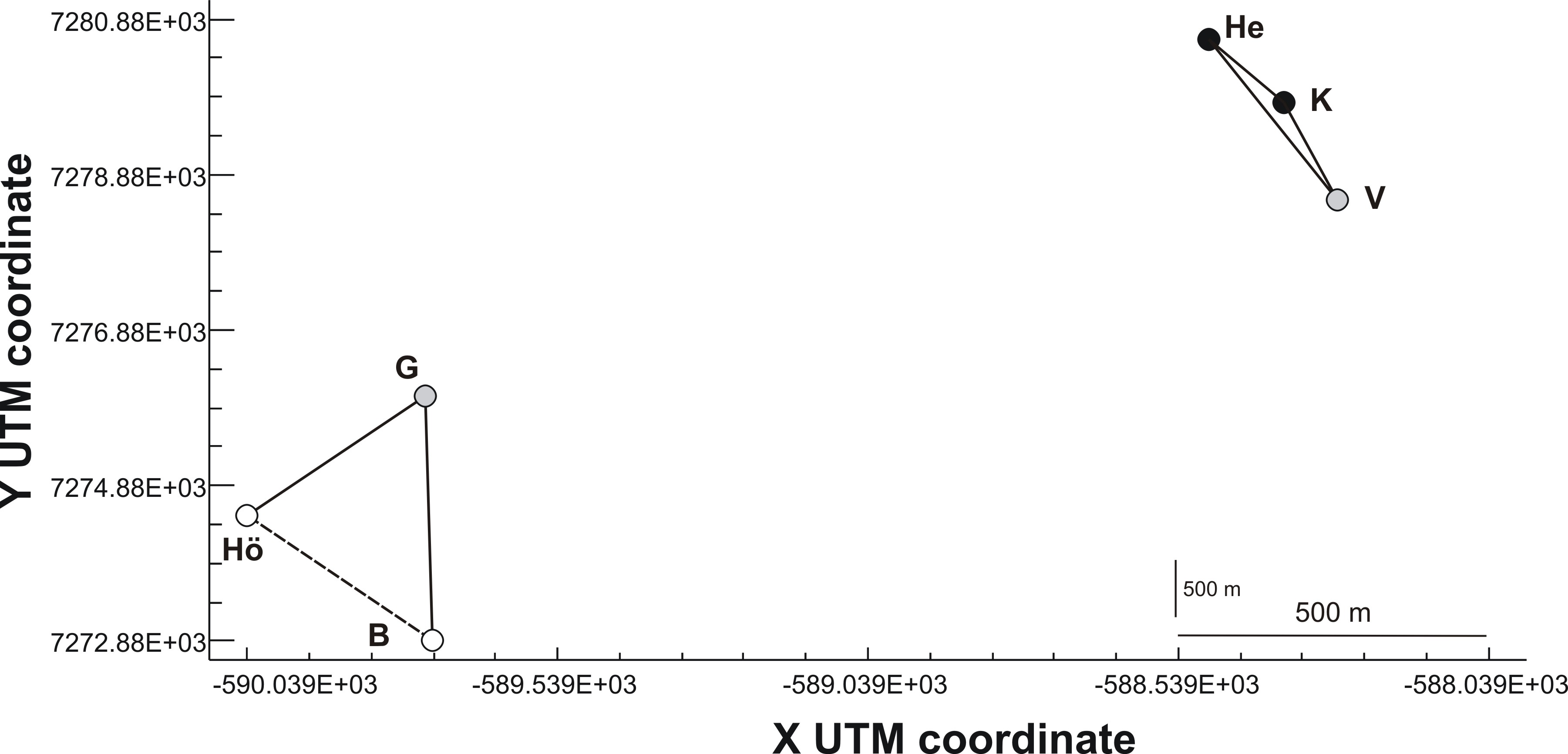 |
| --- |
| b) 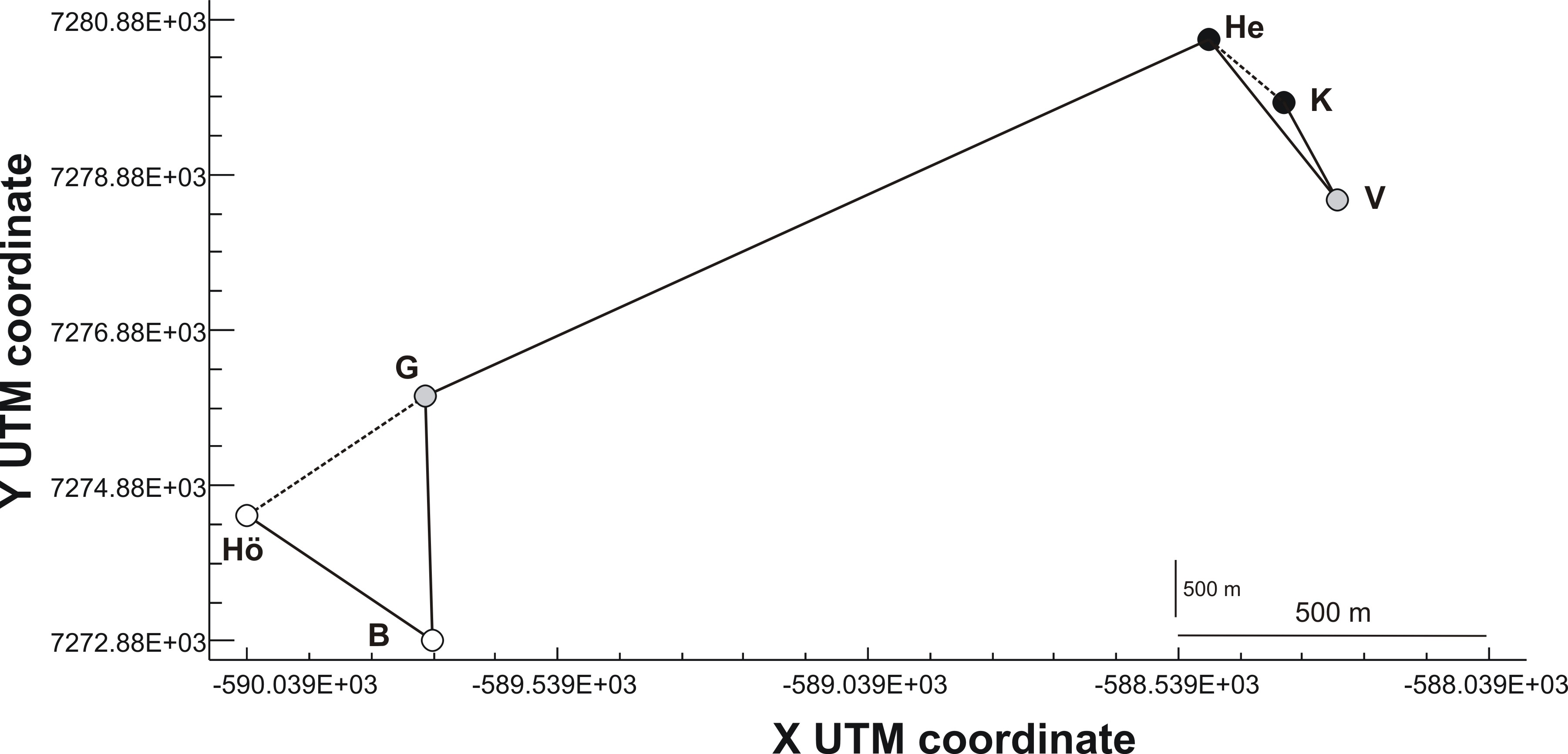 |
| c) 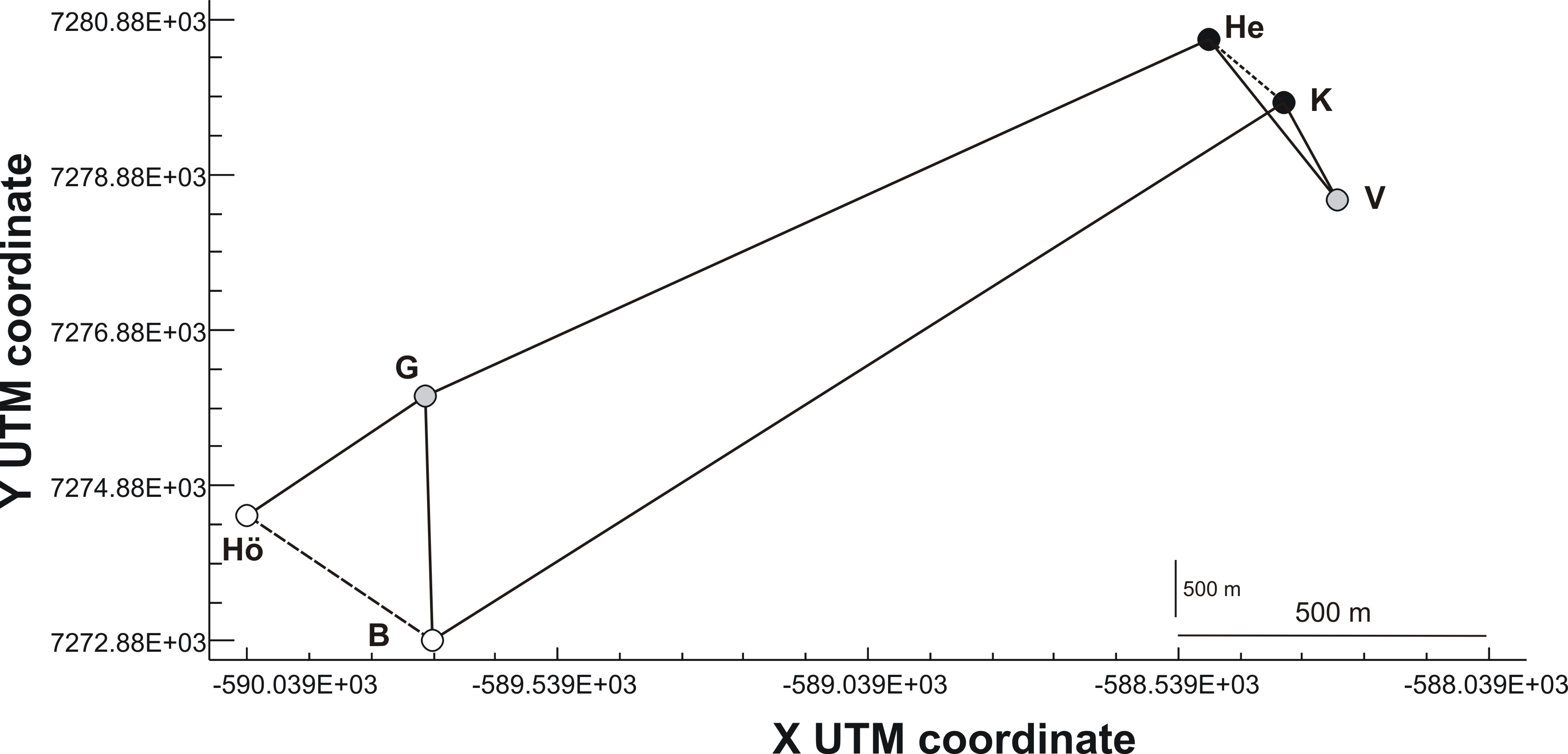 |

Fig. B. Graphs of the AFLPs genetic networks created with a) six loci under directional selection detected by BayeScan with log_10_(BF)>2 (“very strong selection”); b) 60 loci under directional selection detected by SAM, and c) six loci under directional selection detected by Mcheza. Black circles represent populations in high temperature sites (24 ºC), grey circles depict intermediate temperatures (12-19 ºC) and white circles represent sites at low temperature (6-8 ºC). Site pairs in a network connected by lines are considered to exchange migrants exhibiting significant conditional genetic covariance. Solid lines indicate connections assuming an IBD process where genetic distances and spatial distances are proportional. Dotted lines (…) represent compressed edges with relatively higher conditional genetic distance (cGD) in respect to spatial distance (suggesting geographical or ecological barriers), whereas dashed lines (---) depict extended edges indicating long distance migration processes.

| 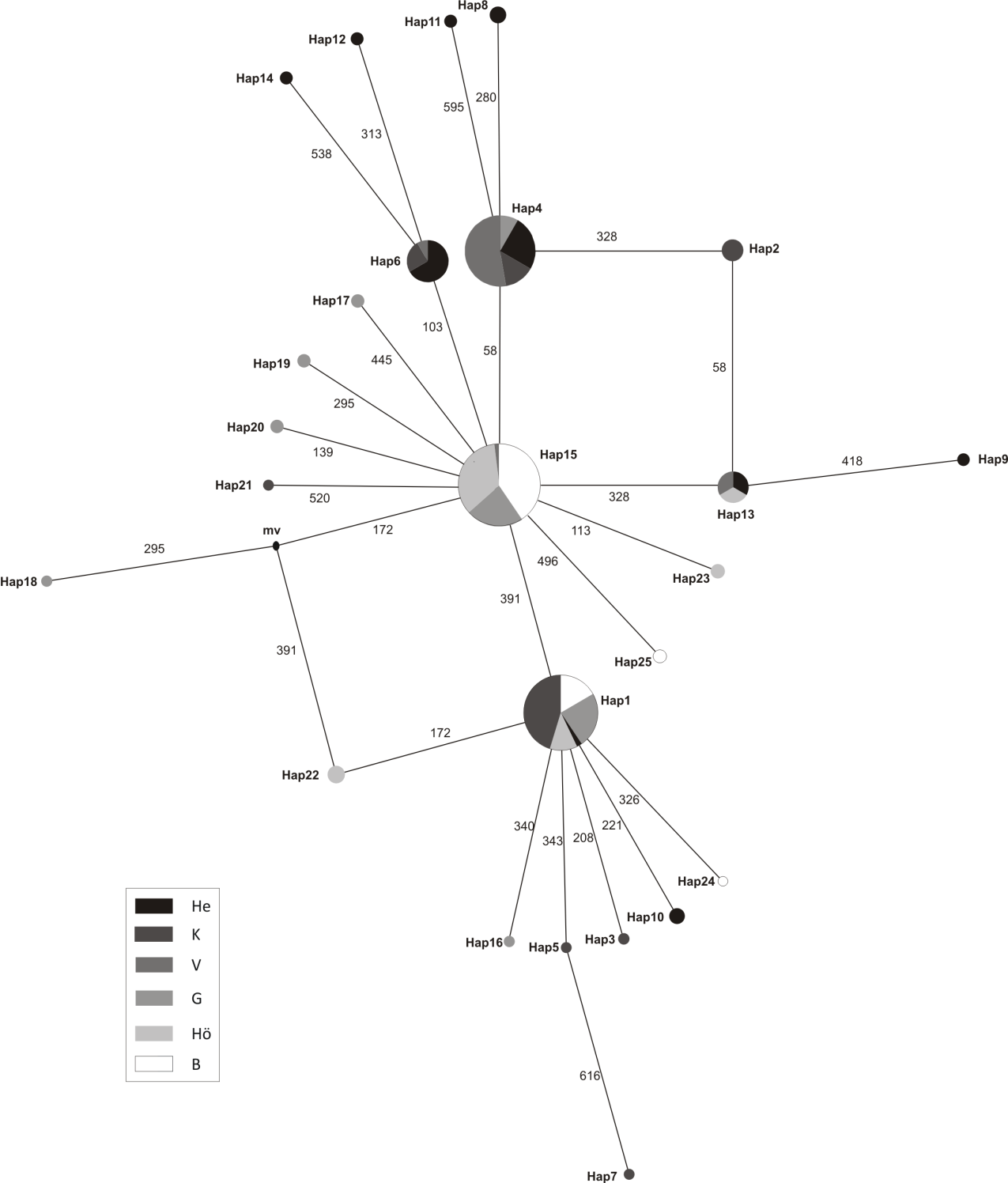 |
| --- |

Fig. C. Median-joining network of COI haplotypes. Each haplotype is represented by a circle, and its area is proportional to its relative frequency; shared haplotypes are represented as frequency diagrams. Smaller black circle (mv) represents an unsampled hypothetical haplotype. Numbers correspond to mutational positions in the studied 643-bp fragment. Colors in the diagrams follow a decreasing thermal trend (*i.e.* warmest populations are depicted in the darkest colors).

TABLES

Table A. Diversity statistics by population for 376 repeatable AFLP markers, using Bayesian estimation of allele frequencies with non-uniform priors [[6](#_ENREF_6)]. Numbers in parenthesis correspond to the values for the 117 neutral loci found simultaneously with BayeScan, Mcheza and SAM.

| **Population** | **Polymorphic loci** | **Polymorphic loci (%)** | **Hj^a^ ± S.E.** | **Var(Hj)** | **Variance component I (%)^b^** | **Variance component L (%)^c^** |
| --- | --- | --- | --- | --- | --- | --- |
| Helgavogur (He) | 223  (107) | 59.3  (91.5) | 0.2534 ± 0.0081  (0.3472 ± 0.0132) | 6.2E-05  (0.00017) | 31.3  (21.7) | 68.7  (78.3) |
| Kalstjörn (K) | 188  (105) | 50  (89.7) | 0.2177 ± 0.0084  (0.3285 ± 0.0142) | 7.3E-05  (0.00020) | 29.1  (20.1) | 70.9  (79.9) |
| Víkurnes (V) | 187  (108) | 49.7  (92.3) | 0.2412 ± 0.0085  (0.3671 ± 0.0126) | 6.6E-05  (0.00016) | 29.3  (21) | 70.7  (79) |
| Geiteyjarströnd (G) | 366  (113) | 97.3  (96.6) | 0.2748 ± 0.0079  (0.3896 ± 0.0112) | 7.6E-05  (0.00012) | 33.7  (24.2) | 66.3  (75.8) |
| Höfði (Hö) | 173  (106) | 46  (90.6) | 0.2300 ± 0.0088  (0.3666 ± 0.0136) | 7E-05  (0.00018) | 26.3  (17.9) | 73.7  (82.1) |
| Bjarnrus (B) | 180  (112) | 47.9  (95.7) | 0.2271 ± 0.0085  (0.3646 ± 0.0121) | 7.1E-05  (0.00015) | 27.8  (25.8) | 72.2  (74.2) |

^a^ Within-population expected heterozygosity, equivalent to Nei's gene diversity.

^b^ Proportion of Hj due to sampling of individuals.

^c^ Proportion of Hj due to sampling of loci.

**Table B.** Shaded cells with numbers in bold depict loci detected to be under directional selection by: SAM (P values for G and Wald Beta 1 with a significance threshold set to 95% corresponding to P< 0.000103093 after Bonferroni correction); BayeScan (log_10_(BF)>1.5 corresponding to “very strong selection”) and MCHEZA at a significance *P* value of 0.01.

| **AFLP Marker** | **SAM** | | **BAYESCAN** | **MCHEZA** |
| --- | --- | --- | --- | --- |
|  | ***P* value for G** | ***P* value for Wald Beta 1** | **log_10_(BF)** | **P (Simul F_ST_<sample F_ST_)** |
| **C21_220** | 0 | 1.06577E-07 | **1.5421** | **0.99550** |
| **C36_295** | 2.66454E-15 | 5.40712E-12 | **1.5982** | 0.854036 |
| **C13_66** | 3.33067E-15 | 3.70057E-10 | **1.6792** | 0.935766 |
| **C83_147** | 0 | 1.11737E-11 | **1.7295** | **0.99550** |
| **C16_156** | **1.55431E-15** | **4.13347E-12** | **2.1645** | **0.99625** |
| **C61_305** | **0** | **1.27097E-09** | **2.5215** | 0.993002 |
| **C34_282** | **0** | **6.93279E-12** | **2.5516** | **0.99625** |
| **C83_137** | **0** | **3.65323E-11** | **3.1546** | 0.990752 |
| **C34_277** | **0** | **4.21885E-14** | **3.6988** | **0.99650** |
| **C18_143** | **0** | **5.56502E-10** | **1000** | **0.99925** |

**Table C. Analysis of four populations at extreme temperatures.-** Shaded cells with numbers in bold depict loci detected to be under directional selection by: SAM (P values for G and Wald Beta 1 with a significance threshold set to 95% corresponding to P< 0.000103093 after Bonferroni correction); BayeScan (log_10_(BF)>1.5 corresponding to “very strong selection”) and MCHEZA at a significance P value of 0.01. The markers selected in the analyses with the total six populations are depicted in bold italics

| **AFLP Marker** | **SAM** | | **BAYESCAN** | **MCHEZA** |
| --- | --- | --- | --- | --- |
|  | ***P* value for G** | ***P* value for Wald Beta 1** | **log_10_(BF)** | **P (Simul F_ST_<sample F_ST_)** |
| ***C18_143*** | 0 | 0.005344848 | **3.5227** | **0.99900** |
| ***C16_156*** | **2.22045E-15** | **9.75132E-11** | **2.1045** | **0.99800** |
| ***C34_277*** | **0** | **8.02569E-12** | **1.7806** | 0.98001 |
| C83_137 | **2.44249E-15** | **6.22007E-10** | **1.5982** | 0.97901 |
| ***C34_282*** | **5.5511E-16** | **1.43809E-06** | 1.4328 | 0.99300 |

**Table D.** Distribution of frequencies (%) per population for the four markers under directional selection detected with the three approaches (BayeScan, Mcheza and SAM) simultaneously.

| **Temperature (ºC)** | **Population** | **AFLP locus** | | | |
| --- | --- | --- | --- | --- | --- |
|  |  | **C18_143** | **C34_277** | **C16_156** | **C34_282** |
| 24º | Helgavogur (He) | 100 | 90 | 100 | 33 |
| 24º | Kalstjörn (K) | 100 | 90 | 57 | 23 |
| 19º | Víkurnes (V) | 93 | 93 | 70 | 33 |
| 12º | Geiteyjarströnd (G) | 60 | 27 | 43 | 87 |
| 8º | Höfði (Hö) | 37 | 10 | 10 | 100 |
| 6º | Bjarnrus (B) | 7 | 3 | 7 | 97 |

**Table E.** Pairwise F_ST_ between populations calculated with AFLPSurv for the 117 neutral data (below diagonal) and pairwise F_ST_ for the four loci under directional selection (above diagonal). Pairwise F_ST_ for loci under stabilizing selection took value of 0.000^NS^ for every pair of populations. Significance was based on 10000 permutations.

|  | **He** | **K** | **V** | **G** | **Hö** | **B** |
| --- | --- | --- | --- | --- | --- | --- |
| **He** | ------------ | 0.2231*** | 0.1621*** | 0.4845*** | 0.7241*** | 0.7740*** |
| **K** | 0.0739*** | ------------ | 0.0065^NS^ | 0.3312*** | 0.5946*** | 0.6543*** |
| **V** | 0.0531*** | 0.0433*** | ------------ | 0.2675*** | 0.5288*** | 0.5843*** |
| **G** | 0.0852*** | 0.0930*** | 0.0523*** | ------------ | 0.1113*** | 0.1629*** |
| **Hö** | 0.1307*** | 0.1275*** | 0.0967*** | 0.0799*** | ------------ | 0.0309** |
| **B** | 0.0967*** | 0.0992*** | 0.0813*** | 0.0818*** | 0.0828*** | ------------ |

Table F. mtDNA. Distribution of frequencies (%) per population for the haplotypes showing correlation with temperature according to SAM.

| **Temperature (ºC)** | **Population** | **mtDNA haplotype** | | |
| --- | --- | --- | --- | --- |
|  |  | **Hap_4** | **Hap_6** | **Hap_15** |
| 24º | Helgavogur (He) | 32 | 29 | 0 |
| 24º | Kalstjörn (K) | 17 | 10 | 0 |
| 19º | Víkurnes (V) | 68 | 4 | 4 |
| 12º | Geiteyjarströnd (G) | 10 | 0 | 40 |
| 8º | Höfði (Hö) | 0 | 0 | 62 |
| 6º | Bjarnrus (B) | 0 | 0 | 70 |

Hap_4: TTGTTCAACGCCTTGCTTATTT

Hap_6: GCGTTCAACGCCTTGCTTATTT

Hap_15: GTGTTCAACGCCTTGCTTATTT

Table G. Pairwise Φ_ST_ between populations calculated with the 22 haplotypes not correlated with temperature (below diagonal) and with the 3 correlated haplotypes (above) computed from haplotype frequencies frequencies with Arlequin. Significance was based on 10000 permutations

|  | **He** | **K** | **V** | **G** | **Hö** | **B** |
| --- | --- | --- | --- | --- | --- | --- |
| **He** | ------------ | 0.0000^NS^ | 0.2970*** | 0.5083*** | 0.6917*** | 0.7583*** |
| **K** | 0.2348*** | ------------ | 0.2020^NS^ | 0.5203*** | 0.7550*** | 0.8444*** |
| **V** | 0.1716^NS^ | 0.0253^NS^ | ------------ | 0.6742*** | 0.8457*** | 0.9025*** |
| **G** | 0.2034*** | 0.0020^NS^ | 0.0000^NS^ | ------------ | 0.0863^NS^ | 0.1845^NS^ |
| **Hö** | 0.0843^NS^ | 0.0571^NS^ | 0.0000^NS^ | 0.0262^NS^ | ------------ | 0.0054^NS^ |
| **B** | 0.2546*** | 0.0002^NS^ | 0.0000^NS^ | 0.0000^NS^ | 0.0554^NS^ | ------------ |

**Table H.** Results on Mantel and partial Mantel tests comparing matrices of geographic distance (Geo), temperature (Temp) and genetic distance (estimated as pairwise Graph Distance with GeneStudio) for AFLPs and COI, and assessed for: neutral markers (NGD); loci under directional selection (DirGD) and loci under stabilizing selection (StaGD). Boldface type indicates significant values after 9999 permutations.

| **Loci** | **Mantel tests** | | | **Partial Mantel tests** | | |
| --- | --- | --- | --- | --- | --- | --- |
|  | **Matrices** | **Mantel´s r** | ***P*-value** | **Matrices** | **Mantel´s r** | ***P*-value** |
| **AFLP markers** |  |  |  |  |  |  |
|  | Geo-NGD | 0.4116 | 0.0617 | Geo-NGD(Temp) | -0.0156 | 0.4632 |
|  | Geo-DirGD | **0.8359** | **0.0074** | Geo-DirGD (Temp) | 0.3428 | 0.2893 |
|  |  |  |  | Geo-DirGD(NGD) | **0.7989** | **0.0095** |
|  | Geo-StaGD | 0.1255 | 0.3315 | Geo-StaGD(Temp) | -0.1912 | 0.2979 |
|  |  |  |  | Geo-StaGD(NGD) | -0.1478 | 0.2964 |
|  | Temp-NGD | **0.4892** | **0.0474** | Temp-NGD(Geo) | 0.2903 | 0.2556 |
|  | Temp-DirGD | **0.8772** | **0.0164** | Temp-DirGD(Geo) | 0.5698 | 0.1318 |
|  |  |  |  | Temp-DirGD(NGD) | **0.8418** | **0.0245** |
|  | Temp-StaGD | 0.2582 | 0.1735 | Temp-StaGD(Geo) | 0.2940 | 0.3122 |
|  |  |  |  | Temp-StaGD(NGD) | -0.0309 | 0.3945 |
| **COI mtDNA** | Geo-NGD | **-0.5726** | **0.0110** | Geo-NGD(Temp) | -0.5222 | 0.1743 |
|  | Geo-DirGD | **0.6961** | **0.0259** | Geo-DirGD(Temp) | 0.1873 | 0.3477 |
|  |  |  |  | Geo-DirGD(NGD) | **0.5946** | **0.0379** |
|  | Temp-NGD | -0.3768 | 0.0642 | Temp-NGD(Geo) | 0.2673 | 0.3059 |
|  | Temp-DirGD | **0.7368** | **0.0420** | Temp-DirGD(Geo) | 0.3797 | 0.1884 |
|  |  |  |  | Temp-DirGD(NGD) | **0.6851** | **0.0461** |
